# Ginsenoside Re ameliorates thioacetamide-induced acute liver injury through inhibiting autophagy-NLRP3 inflammasome pathway

**DOI:** 10.3389/fphar.2025.1592203

**Published:** 2025-06-20

**Authors:** Jing Lin, Huan Wang, Ruowei Zhao, Shaohua Li, Dennis Chang, Yanfang Zheng, Xian Zhou, Rui Huang, Mingqing Huang

**Affiliations:** ^1^ The Affiliated People’s Hospital, College of Pharmacy, Fujian University of Traditional Chinese Medicine, Fuzhou, China; ^2^ NICM Health Research Institute, Western Sydney University, Westmead, NSW, Australia; ^3^ Department of Nursing, Mengchao Hepatobiliary Hospital of Fujian Medical University, Fuzhou, Fujian, China

**Keywords:** ginsenoside Re, acute liver injury, PI3K/AKT/mTOR, autophagy, NLRP3 inflammasome, male mice

## Abstract

**Background:**

Ginsenoside Re (G-Re), a unique ginsenoside almost exclusively found in Araliaceae plants, is a promising therapeutic agent for attenuating liver injury. This study aims to investigate the liver-protective effects of G-Re and the underlying mechanisms in acute liver injury models.

**Methods:**

Male C57BL/6 mice were intraperitoneally injected with various agents induce the acute liver injury model after pre-treatment with G-Re (5–20 mg/kg, oral gavage). Additionally, the phosphoinositide 3-kinases (PI3K) inhibitor LY294002 and the mammalian target of rapamycin (mTOR) inhibitor RAPA were co-administered with G-Re in the thioacetamide (TAA)-induced rat hepatic stellate cell line (HSC-T6) to explore the mechanisms associated with G-Re.

**Results:**

G-Re at (20 mg/kg) protected liver against thioacetamide (TAA), ethanol, acetaminophen, and D-Galactosamine-induced liver injury in C57BL/6 mice. G-Re reduced serum levels of aspartate aminotransferase (AST) from 151.98 to 40.24 U/L and alanine aminotransferase (ALT) from 392.04 to 49.43 U/L. Both *in vivo* and *in vitro* studies consistently showed that G-Re decreased mRNA expression levels of key pro-inflammatory cytokines, including tumor necrosis factor-α (TNF-α) and interleukin-1β (IL-1β). Additionally, G-Re dose-dependently downregulated the protein expression of cyclooxygenase-2 (COX-2), inducible nitric oxide synthase (iNOS), NOD-like receptor protein 3 (NLRP3), cysteinyl aspartate specific proteinase −1 (caspase-1), interleukin-18 (IL-18), and IL-1β. In addition, our results suggested that the suppression of autophagy by G-Re may play a crucial role in its ability to inhibit the NLRP3 inflammasome. Notably, this regulatory effect on autophagy appears to be mediated through the phosphatidylinositide 3-kinases/protein kinase B/mammalian target of rapamycin (PI3K/AKT/mTOR signaling pathway). G-Re inhibits autophagy in both cellular and animal models by downregulating the expression of light chain 3-II (LC3-II), Beclin-1, and sequestosome-1 (p62) through this pathway. Furthermore, the PI3K inhibitor LY294002 and the mTOR inhibitor rapamycin (RAPA) were shown to partially reverse the inhibitory effects of G-Re on autophagy and inflammation in HSC-T6 cells. These results further support the notion that reactivation of autophagy can counteract G-Re–mediated suppression of NLRP3 and caspase-1 expression.

**Conclusion:**

This study highlights G-Re as a promising therapeutic candidate for liver injury, acting through inhibition of autophagy and inflammation via the PI3K/AKT/mTOR signaling pathway.

## 1 Introduction

Acute liver injury (ALI), characterized by rapid hepatocyte degeneration, necrosis, and apoptosis, can result from viral infections, drug overdose, alcohol intake, or environmental toxins (Katarey and Verma, 2016; Imperatrice et al., 2019). The incidence of ALI continues to rise globally and has become the second most common form of liver injury after hepatitis (Stracieri and Scarpelini, 2006). Drug-induced liver injury (DILI) accounts for a significant portion of ALI cases, contributing to 15%–30% of fulminant hepatic failure and up to 50% of non-viral chronic hepatitis (Tansel et al., 2015). If unresolved, ALI may progress to liver fibrosis, cirrhosis or hepatocellular carcinoma (Negro, 2021; Garrido and Djouder, 2021).

Experimental models using hepatotoxins like thioacetamide (TAA), carbon tetrachloride (CCl_4_), D-galactosamine (D-gal), alcohol (Acol), and acetaminophen (APAP), are widely used to investigate liver injury mechanisms (Liu et al., 2017). TAA, in particular, induces oxidative stress and inflammation through cytochrome P450 2E1-mediated bioactivation, serving as a well-established model of acute and chronic liver damage (Dodge et al., 2006).

Recent studies highlight the critical role of the NOD-like receptor protein 3 (NLRP3) inflammasome in sterile liver inflammation (Zou et al., 2021). Activation of NLRP3 inflammasome, via the Nuclear factor kappa-light-chain-enhancer of Activated B cells (NF-κB) signaling pathway leads to the cleave and release of pro-inflammatory cytokines interleukin-1β (pro-IL-1β)and pro-interleukin-18 (pro-IL-18), contributing to liver injury progression (Al Mamun et al., 2020). Inhibiting the NLRP3 inflammasome has shown protective effects in both acute and chronic liver injuries models.

Autophagy, a cellular mechanism for recycling damaged organelles and proteins, also play a regulatory role in inflammation. (Mizushima et al., 2008). Dysregulation of autophagy has been implicated in the pathogenesis of liver injury.

Ginsenoside Re (G-Re), a major bioactive compound from *Panax notoginseng* (Burk.) F. H. Chen or *Panax ginseng* C. A. Mey, has demonstrated therapeutic effects in metabolic and inflammatory diseases including diabetes, cardiovascular disorders, and non-alcoholic fatty liver disease (NAFLD) (Gao et al., 2022). These effects are mediated by pathways such as phosphatidylinositol 3-kinase (PI3K)/protein kinase B (AKT) and toll-like receptor 4 (TLR4)/NF-κB, and peroxisome proliferator-activated receptor gamma (PPARγ) (Jiang et al., 2021). However, its role in ALI, particular in relation to the NLRP3 inflammasome and autophagy, remains unclear.

This study screened the hepatoprotective efficacy of G-Re across five acute liver injury models (TAA, alcohol, acetaminophen, D-galactosamine, and CCl_4_). G-Re demonstrated significant liver protective effect in all models except CCl_4_-induced liver injury. Based on this screening, we selected the effective TAA model to investigate G-Re’s hepatoprotective effects in depth and explore its underlying mechanisms, focusing on the modulation of NLRP3 inflammasome activity and autophagy via the PI3K/AKT/mTOR signaling pathway. To our knowledge, this is the first comprehensive evaluation of G-Re’s role in regulating autophagy-inflammasome crosstalk in acute liver injury models.

## 2 Materials and methods

### 2.1 Regents and chemicals

Ginsenoside Re (G-Re, Catalog No. S27663) was purchased from Shanghai Yuanye Bio-Technology Co., Ltd., China. Diammonium glycyrrhizinate (DG, Batch No. 210107402) was obtained from Zhengda Tianqing Pharmaceutical Group Co., Ltd., China. Assay kits for aspartate aminotransferase (AST, Catalog No. C010-2-1), alanine aminotransferase (ALT, Catalog No. C010-2-1), and glutathione S-transferase (GST, Catalog No. C010-2-1) were acquired from Nanjing Jiancheng Bioengineering Institute, China. Lipopolysaccharide (LPS, Catalog No. L8880), hematoxylin-eosin (H&E) staining kit (Catalog No. G1120) were sourced from Beijing Solarbio Science and Technology Co., Ltd., China. Rapamycin (Catalog No. HY-10219) was purchased from MedChemExpress (MCE), United States. The Reverse Transcription Kit (Catalog No. K1691), Dulbecco’s Modified Eagle Medium (DMEM medium, Catalog No. C11995500BT) and fetal bovine serum (Catalog No. 26010-074) were obtained from Thermo Fisher Scientific, United States. Anti-(cyclooxygenase-2) (COX, 212375-1-AP) and inducible nitric oxide synthase (iNOS, 18985-1-AP), secondary antibodies including HRP-conjugated Goat Anti-Mouse IgG (H + L) (SA00001-1) and HRP-conjugated Goat Anti-Rabbit IgG (H + L) (SA00001-2) were obtained from Proteintech. β-actin was obtained from TransGen Biotech, China (HC201). Antibodies specific for interleukin-1β (IL-1β, ab283818), phosphoinositide 3-kinases (PI3K, ab154598), phosphorylated-PI3K (p-PI3K, ab278545), NOD-like receptor protein 3(NLRP3, ab263899), and interleukin-18 (IL-18, ab207323) were purchased from Abcam plc, United States. Antibodies targeting sequestosome-1 (P62, 23214S), light chain 3 I/II (LC3I/II, 12741S), protein kinase B (AKT,4691S), phosphorylated-AKT (p-AKT, 4060S), unc-51 like autophagy activating kinase 1 (ULK1, 8054S), phosphorylated-ULK1 (p-ULK1, 14202S), mammalian target of rapamycin (mTOR, 2983S), phosphorylated-mTOR (p-mTOR, 5536S), autophagy-related protein 7 (ATG7, 8558S), autophagy-related protein 5 (ATG5, 12994S), and Beclin-1 (3738S) were obtained from Cell Signaling Technology, Inc., United States. Caspase-1 antibody (sc-12742) was procured from Santa Cruz Biotechnology, Inc., United States.

### 2.2 Animals

Male C57BL/6 mice (20 ± 2 g, 13 weeks old) were purchased from Shanghai Slac Laboratory Animal Co. Ltd (Shanghai, China). All animal experiments were conducted in compliance with the ARRIVE guidelines (Percie du Sert et al., 2020). The mice were housed at the Experimental Animal Center of Fujian University of Traditional Chinese Medicine [SYX: (Min) 2019-0007], with free access to food and water, under a 12 h light/dark cycle. Prior to conducting the experiments, the protocols involving animal subjects received approval from the Animal Care and Use Committee of the Fujian University of Traditional Chinese Medicine (Approval No.: FJTCM IACUC2021068).

### 2.3 Experimental design

Animal experiments utilizing multiple models of ALI were conducted using male C57BL/6 mice of SPF grade, which were randomly divided into 11 groups (n = 6 mice per group, totaling 66 mice). The groups were designated as follows: Control group (Con), TAA group (50 mg/kg, TAA), 50% alcohol group (12 mL/kg, Acol), acetaminophen group (480 mg/kg, APAP), D-galactosamine group (480 mg/kg, D-gal), 10% carbon tetrachloride group (10 mL/kg, CCl_4_), and five G-Re treatment groups: TAA + G-Re group, Acol + G-Re group, APAP + G-Re group, D-gal + G-Re group, and CCl_4_ + G-Re group, with the G-Re dose set at 20 mg/kg. Except for the Con group and the model groups, the G-Re groups received daily gavage (i.g.) once a day for three consecutive days. The Con group and the model groups were given an equivalent volume of saline. 2 h after the last administration, all groups except the Con group were intraperitoneally injected (i.p.) with TAA or CCl_4_, oral gavagewith Acol, APAP, or D-gal. Tissues were collected 16 h after the completion of the modeling process (Table 1).

For TAA-induced liver injury experiment, male C57BL/6 mice with SPF-grade were randomly divided into six groups (n = 6 mice per group, totaling 36 mice). The groups were designated as follows: Control group (Con), Model group (TAA), Positive drug group with 30 mg/kg DG, low-dose G-Re group (5 mg/kg, G-Re-L), medium-dose G-Re group (10 mg/kg, G-Re-M), and high-dose G-Re group (20 mg/kg, G-Re-H). Oral gavage was administered once a day for three consecutive days to all groups except the Con group and TAA groups, which received an equivalent amount volume of saline. Two hfollowing the last administration, all groups except the Con group were intraperitoneally injected with TAA at a dose of 50 mg/kg (Ezhilarasan, 2023). The Con group received intraperitoneal injection of an equivalent volume of saline. Tissues were collected 16 h after the modeling process.

### 2.4 Measurement of serum transaminase enzymes and blood lipids

Blood samples were obtained through eyeball extraction and allowed to coagulate fully at room temperature for 1 h. Subsequently, the blood was subjected to centrifugation at 3000 rpm for 10 min at 4°C. The resulting supernatant containing serum was collected for the assessment of AST, ALT, GST levels. To measure ALT and AST levels, 5 μL of the supernatant was reacted with the relevant reagents for 30 min, followed by reaction with 2,4-dinitrophenylhydrazine and further incubation under alkaline conditions for 15 min. The optical density (OD) was then measured at 505 nm. For GST level detection, 100 μL of the sample was first mixed with the substrate solution and centrifuged. The supernatant was then reacted with the appropriate reagents for 15 min, and the OD value was measured at 412 nm (Lin et al., 2014).

### 2.5 Pathological staining of liver tissue

Liver tissues were initially collected and fixed in polyformaldehyde for 24 h. A portion of the liver tissue was subsequently transferred to embedding molds. The tissue underwent a dehydration process involving 75%, 85% and 95% ethanol. Afterward, it was thoroughly cleaned using xylene solutions and embedded in wax. The wax-embedded section was then sliced to a thickness of 4–6 µm and dried through heating. Following this, the waxed section underwent another round of cleaning with xylene solutions and was dried for 8 h at 50°C, followed by rehydration with descending ethanol. The dewaxed section was then stained with hematoxylin solution for 10 min Excess stain was immersed in a differentiation solution for 3 min. Finally, the dewaxed section was placed in eosin staining solution and stained for 30 s, followed by a brief rinse of 2–3 s with distilled water. After staining, the tissue sections were dehydrated and cleared again, then mounted with neutral resin. Once the resin was fully dried, pathological changes were observed under a light microscope. For each group, tissue sections were prepared from three animals, with one section per animal. Three representative areas were captured from each section using the microscope (Wang et al., 2022).

### 2.6 LPS induced liver injury and drug treatment

Rat hepatic stellate (HSC-T6) cells were sourced from the Kunming Cell Bank of the Chinese Academy of Sciences. To prepare a stock solution of G-Re, it was dis-solved in the DMEM culture medium containing 20% dimethyl sulfoxide (DMSO) to prepare a stock solution with a concentration of 40 μM. HSC-T6 cells were seeded in a 6-well plate and allowed to grow for 36 h. Subsequently, they were treated with G-Re at concentrations of 10 μM, 50 μM or 100 μM, either with or without LPS stimulation of 100 ng/mL. In addition, a group of cells treated with G-Re at 100 μM was co-incubated with the mTOR inhibitor RAPA at 20 μM, or the PI3K inhibitor LY294002 at 20 μM. The cells were then further incubated for a period of 12 h, after which protein extraction was carried out.

### 2.7 MTT for cell viability

HSC-T6 cells were seeded in a 96-well plate and allowed to grow for 36 h. The cells were then treated with G-Re at different concentrations (0, 5, 10, 20, 50, 100, 150, and 200 μM) for 24 h. MTT solution (0.5 mg/mL) was added to the plate and allowed to incubate for an additional 4 h. The supernatant was then replaced with DMSO (100 μL per well). The plate was placed on a shaker and agitated for 10 minto ensure complete dissolution, and the ODwas measured at a wavelength of 490 nm.

### 2.8 PCR

A 100 mg sample of stored liver tissue was finely chopped into small pieces and mixed with 1 mL of RNA isolator in a 1:10 volume ratio. After placing on ice for approximatley 15 min, total mRNA was extracted by adding 200 μL of chloroform, followed by 500 μL of isopropanol. The supernatant was discarded, and 500 μL of 75% ethanol was added and thoroughly vortexed. After a 5min centrifugation and three washing steps, the pellet was resuspended in a 50–100 μL of RNase-free water, resulting in the total RNA solution for the experiment. The purity of the total RNA solution was examined by measuring the A260/A280 ratio which was within the range of 1.9–2.1 to guarantee the reliability of subsequent PCR data. A UV spectrophotometer was used to determine the RNA concentration and purity. A total of 2000 ng of RNA sample was transferred into a 200 μL RNase-free EP tube, and the reverse transcription process was carried out following the instructions of the reverse transcription kit using a PCR cycler. The reaction conditions were set at 25°C for 5 min and 42°C for 60 min. The cDNA sample (1 μL) was mixed with 2×ChamQ SYBR qPCR Master Mix (4 μL, Vazyme, Nanjing, China), 50 × ROX Reference Dye 1 (1 μL), diluted primers (2 μL, Fuzhou Watson Biotechnologies Co., Ltd.) and RNase-free ddH_2_O (2 μL). The primer sequences are listed in Table 2. The samples were then subjected to qRT-PCR, following these cycling conditions: step 1, 95°C for 30 s; step 2, 40 cycles at 95°C for 10 s, and 60°C for 30 s and followed by 95°C for 15 s; step 3, 60°C for 60 s and 95°C for 15 s. Each assay was conducted in triplicate from three separate biological experiments and normalized to β-actin expression which served as the internal reference gene. The relative expression levels of various mRNA indicators were calculated using the 2^^−ΔΔCt^ method (Schmittgen and Livak, 2008). The formula for calculating -ΔΔCt is as follows: ΔΔCt = Experimental Group (ΔCt Target Gene - ΔCt β-actin) - Sham Group (ΔCt Target Gene - ΔCt β-actin).

**TABLE 1:** Grouping scheme for multiple ALI models.

Control group	Model groups	Treatment groups
Saline	i.p. TAA (50 mg/kg)	TAA + i.g. G-Re (20 mg/kg)
	i.g. Acol (12 mL/kg)	Acol + i.g. G-Re (20 mg/kg)
	i.g. APAP (480 mg/kg)	APAP + i.g. G-Re (20 mg/kg)
	i.g. D-gal (480 mg/kg)	D-gal + i.g. G-Re (20 mg/kg)
	i.p. CCl_4_ (10 mL/kg)	CCl_4_ + i.g. G-Re (20 mg/kg)

**TABLE 2:** Primer nucleotide sequence list.

Primers	Forward (5'→3′)	Reverse (5'→3′)
TNF-α	GCC​GAT​GGG​TTG​TAC-CTT​GT	TCT​TGA​CGG​CAC​AGA​G-GAG​G
IL-1β	GAA​ATG​CCA​CCT​TTT-GAC​AGT​G	TGG​ATG​CTC​TCA​TCA​G-GAC​AG
IL-6	CTG​CAA​GA-GAC​TTC​CAT​CCA​GCT	AGT​GGT​ATA​GA-CAG​GTC​TGT​TGG
β-actin	GAG​CG-CAA​GTA​CTC​TGT​GTG	AAC​GCA​GCT​CAG-TAA​CAG​TC

### 2.9 Western blot analysis

A portion of the liver tissue collected from each group was homogenised and kept on ice. The liver tissues were combined with 0.5 mL ice-cold RIPA lysis buffer (Beyotime Biotechnology, China) and 1% protease/phosphatase inhibitor cocktail (Solarbio, Beijing, China) on ice for 30 min, with intermittent vertexing every 5–10 min. Subsequently, the cell lysates were subjected to centrifugation at 12,000 rpmfor 10 min. The resulting supernatant was collected, and protein concentration was determined using the BCA Protein Assay Kit (Beyotime Biotechnology, Haimen, China). The total protein samples from each group, at a concentration of 6 μg/μL, were mixed with 5X protein loading buffer (Beyotime Biotechnology, Haimen, China) and denatured at 100°C for 10 min. The proteins were then separated via SDS-PAGE electrophoresis using the Power Pac Basic system (BIORAD, South Granville, Australia). Electrophoresis was conducted at 30V for 0.5h, followed by 90V for 1.5 h in protein gels prepared from the kit (10-well, Beyotime Biotechnology, Haimen, China). Afterward, the proteins were transferred onto PVDF membranes (Millipore, Burlington, United States) before beinging incubated with 5% skim milk dissolved in TBS-T [1 X Tris-buffered saline, 0.1% tween 20 (Thermo Fisher Scientific, North Ryde, Australia)] for 90 min at room temperature. The membranes were incubated overnight at 4°C with the following primary antibodies: anti-COX-2 (1:1000), anti-iNOS (1:5000), anti-NLRP3 (1:100), anti-Caspase-1 (1:500), anti-IL-1β (1:1000), anti-IL-18 (1:1000), anti-p-PI3K (1:1000), anti-PI3K (1:1000), anti-p-AKT (1:1000), anti-AKT (1:1000), anti-p-mTOR (1:1000), anti-mTOR (1:1000), anti-p-ULK1 (1:1000), anti-ULK1 (1:1000), anti-LC3A/B (1:1000), anti-P62 (1:1000), anti-ATG7 (1:1000), anti-ATG5 (1:1000), and anti-Beclin-1 (1:1000). Following three washes with TBS-T buffer, the membranes were immersed in anti-rabbit antibodies conjugated with horseradish peroxidase (1:5000) or anti-mouse conjugated with horseradish pe-roxidase (1:5000) for 1.5 h β-actin (1:5000) served as the internal control. Immunoreactive bands on the membranes were visualized by adding 1 mL of the mixed reagents A and B from the ECL kit (Beyotime, Beijing, China, cat. no. P0018A) using ChemiDoc XRS+ (BioRad, Hercules, United States). Specific bands were analysed, and the intensity was quantified using Image Lab 6.0 software.

### 2.10 Immunofluorescence

The waxed liver section was sliced to a thickness of 4 μm, followed by dewaxing and rinsing with water. It was then subjected to incubation with a goat serum blocking solution at room temperature for 20 min to minimize background intensity. After staining, sections were dehydrated, cleared, and mounted with neutral resin. Once dried, pathological changes were examined under a light microscope. For each group, one section per animal (n = 3) was prepared, and three representative fields were captured per section. The slides were incubated overnight at 4°C with primary antibody LC3II (1:100). The next day, after washing with PBS, they were incubated with HRP-conjugated goat anti-rabbit IgG (1:100) for 20 min at room temperature in the dark. Nuclei were stained with DAPI for 5–10 min at room temperature, air-dried, and then mounted with neutral resin. For each group, liver tissues were collected from three randomly selected animals, with one section prepared per animal. Using an Olympus microscope (BX51T-PHD-J11, Japan), three random fields were observed for positive cells, and results were analyzed with Image Pro Plus software.

### 2.11 Statistical analysis

All data were analyzed using SPSS 26.0 and expressed as mean ± standard error of the mean (SEM). Data normality was assessed by the Shapiro-Wilk test. For normally distributed data, one-way ANOVA was used for statistical analysis; for non-normally distributed data, the nonparametric Mann-Whitney U test was applied. A p-value <0.05 was considered statistically significant.

## 3 Results

### 3.1 *In vivo* study

#### 3.1.1 The protective effects of G-Re in multiple agent-induced acute liver injury models

**FIGURE 1:**
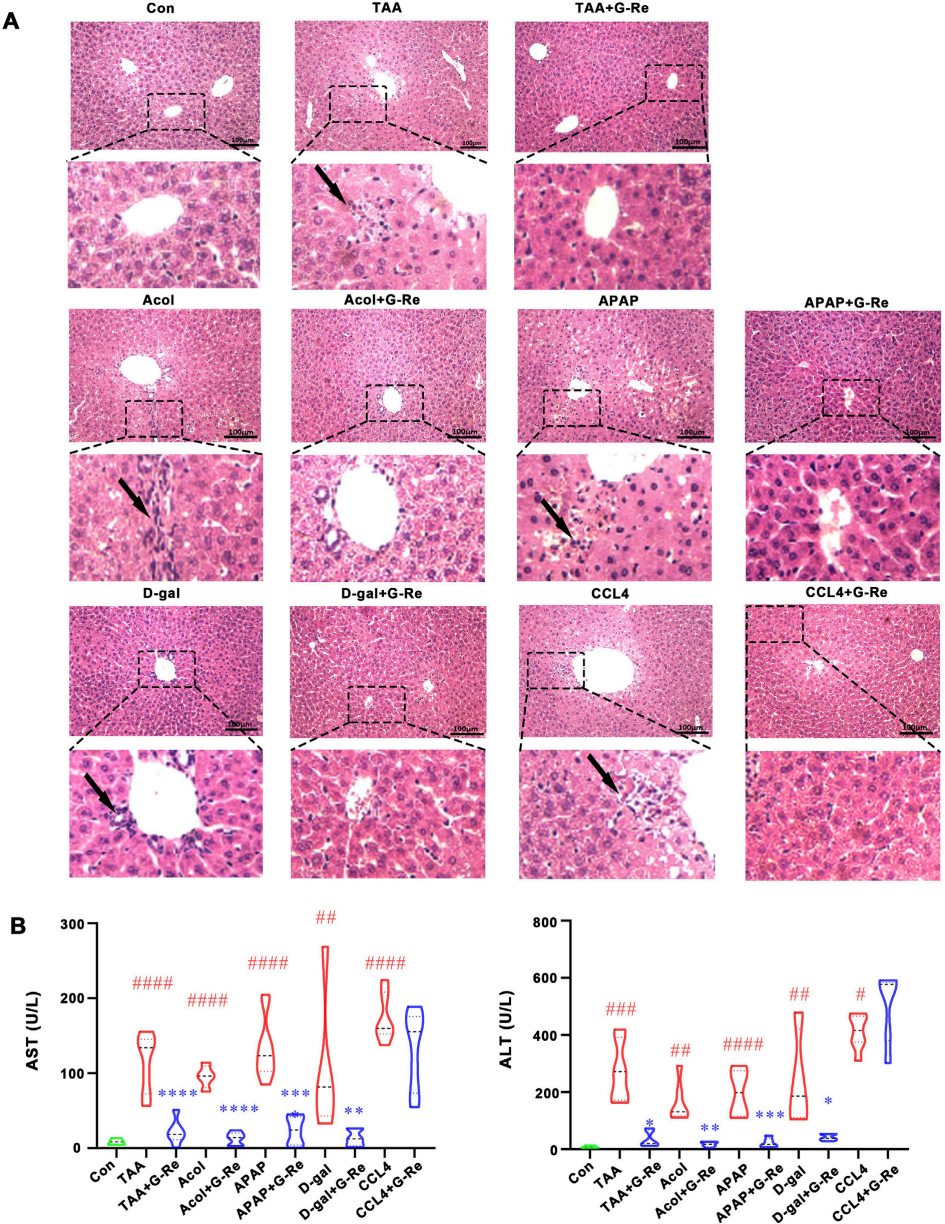
G-Re alleviated acute liver injury in multiple mouse models. **(A)** H&E staining of liver tissue in the control (Con) group, and in various agents induced liver injury models including alcohol (Acol), acetaminophen (APAP), D-galactosamine (D-gal) and CCL_4_ interventions. **(B)** The changes on aspartate aminotransferase (AST) and alanine aminotransferase (ALT) levels (n = 6). #*p* < 0.05, ##*p* < 0.01, ###*p* < 0.001, ####*p* < 0.0001 vs. Con group; *p < 0.05,**p < 0.01, ***p < 0.001, ****p < 0.0001 vs. the corresponding liver injury model group.

In this study, multiple agent-induced acute liver injury models were used to evaluate G-Re’s protective effects. H&E staining images (Figure 1A) revealed that the hepatic lobule structure in the control group was normal, with cells arranged tightly and no necrosis or inflammatory infiltration observed. After TAA intervention, the cells were disorganized with severe congestion in the central vein and hepatic sinusoids, accompanied by inflammatory cell infiltration (indicated by arrows). Similar pathologic changes were also observed after alcohol, acetaminophen, D-galactosamine and CCl_4_ interventions. Following G-Re (20 mg/kg) pre-treatment, hepatocytes appeared relatively normal, with tight arrangement, reduced dilation of the central vein, and improvement in the congestion of the hepatic sinusoid region in mice induced by TAA, alcohol, acetaminophen, and D-galactosamine but not by CCl_4_.

Consistent with H&E staining results, the levels of AST and ALT in the serum of mice induced by TAA, alcohol, acetaminophen, and D-galactosamine were significantly increased, and G-Re significantly reduced their expression levels; however, G-Re had no significant effect on the levels of AST and ALT in the serum of mice induced by CCl_4_ (Figure 1B). Consequently, we selected the effective TAA model for subsequent investigation of hepatoprotective effects and mechanisms.

#### 3.1.2 Hepatoprotective effects of G-Re in mice with TAA-induced acute liver injury

**FIGURE 2:**
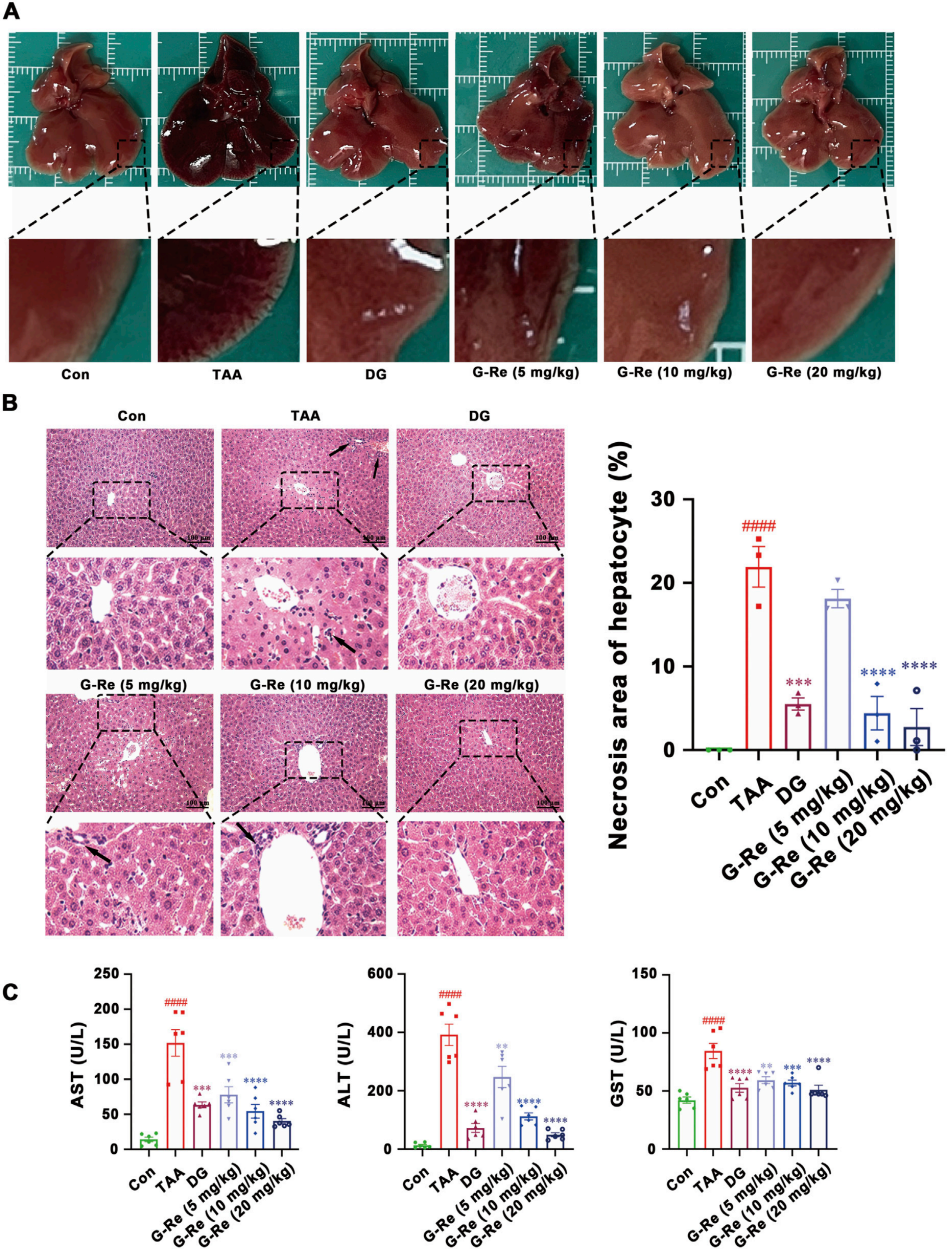
G-Re mitigates TAA-induced liver injury in mice. **(A)** Macroscopic changes of liver tissue. **(B)** H&E staining of liver tissue (n = 3). **(C)** The changes on AST, ALT and GST levels (n = 6). ####*p* < 0.0001 vs. Con group; **p < 0.01, ***p < 0.001, ****p < 0.0001 vs. TAA group.

To further investigate the hepatoprotective activity of this compound, there doses of G-Re (10, 20, 40 mg/kg) were administrated in TAA-induced acute liver injury mice. The liver surface in the vehicle control group appeared to have a smooth appearance, characterized by a soft and resilient texture. In contrast, the livers in the TAA group displayed a dark red color, extensive congestion, and relatively hard texture. However, pre-treatment with DG and G-Re partially ameliorated those observed phenomena (Figure 2A).

H&E staining images revealed that liver cell morphology in the control group was normal, featuring a tightly organized arrangement, well-defined hepatic lobules and sinusoidal structures, and liver cells radiating around the central vein in a radial pattern. Following TAA intervention, there was disordered cell arrangement, severe congestion in the central vein and sinusoids, and infiltration of inflammatory cells (indicated by arrows). Additionally, white fat vacuoles appeared around the liver lobules, accompanied by widespread liver cell nuclear condensation and necrosis. Nevertheless, pre-treatment with DG and G-Re (10, 20 mg/kg) resulted in some improvement in liver cell necrosis and inflammatory infiltration (Figure 2B).

Intraperitoneal injection of TAA significantly elevated the levels of AST, ALT, and GST in the serum of mice (all *p* < 0.0001) compared to the control group (Figure 2C). Conversely, pre-treatment with DG markedly reduced the expression levels of AST, ALT, and GST in the serum of mice compared to the model group (all *p* < 0.0001). Similarly, pre-treatment with G-Re (5, 10, 20 mg/kg) significantly decreased the expression levels of AST, ALT, and GST in the serum of mice, exhibiting a dose-dependent effect.

#### 3.1.3 Anti-inflammatory effects of G-Re in a TAA-induced mouse model of liver injury

**FIGURE 3:**
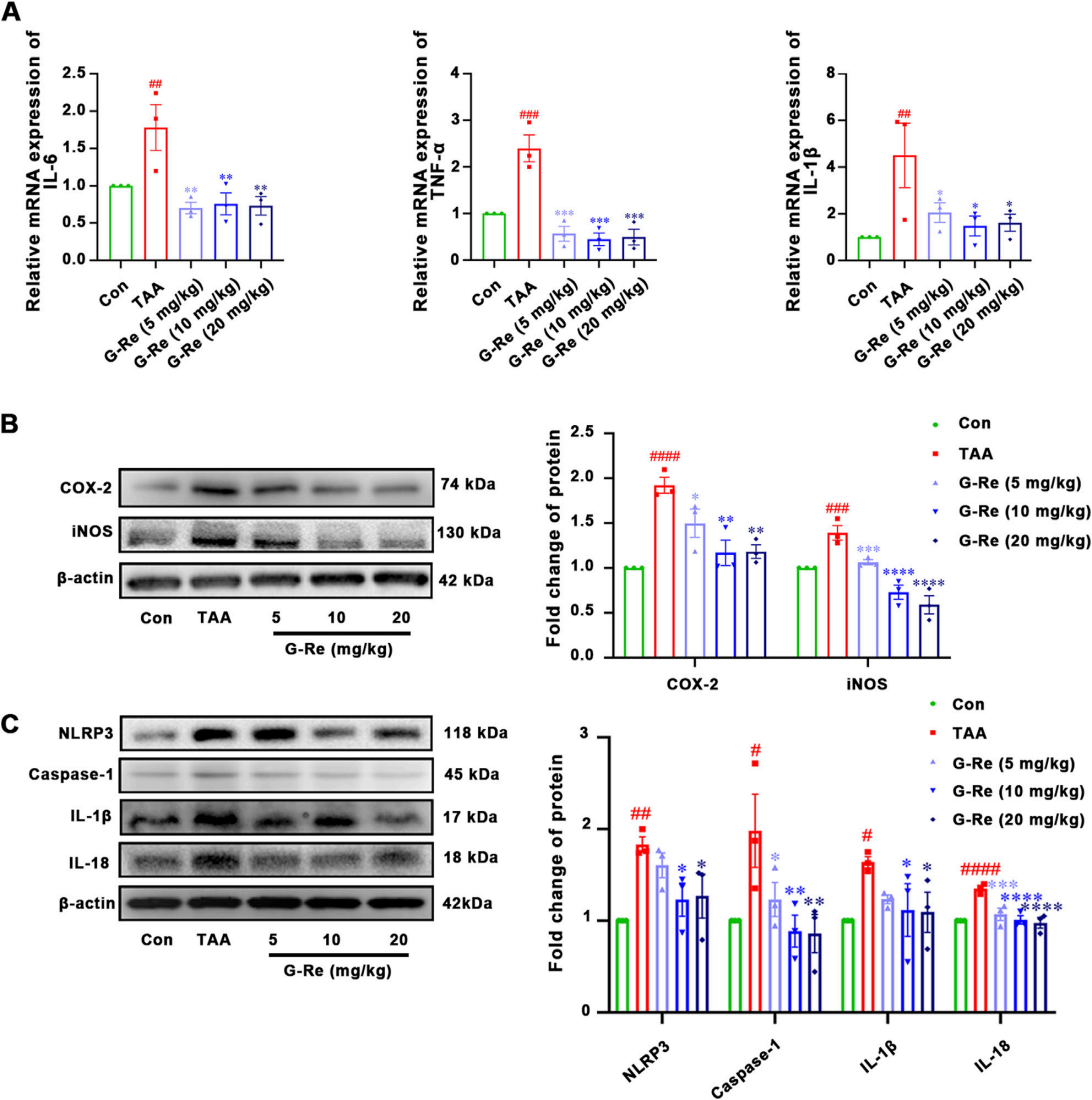
G-Re attenuates inflammatory markers in mice with TAA-Induced Liver Injury (n = 3). **(A)** mRNA expressions of IL-6, TNF-α, and IL-1β. **(B)** The changes on COX-2 and iNOS protein ex-pressions. **(C)** The changes on NLRP3, Caspase-1, IL-1β and IL-18 protein expressions. #*p* < 0.05, ##*p* < 0.01, ###*p* < 0.001, ####*p* < 0.0001 vs. Con group; *p < 0.05, **p < 0.01, ***p < 0.001, ****p < 0.0001 vs. TAA group.

After 16 h of TAA stimulation, there were significant increases in the mRNA levels of interleukin (IL)-6, tumor necrosis factor (TNF)-α, and IL-1β in the mouse liver. However, in comparison to the model group, pre-treatment with varying doses of G-Re significantly reduced the mRNA levels of IL-6, TNF-α, and IL-1β in the mouse liver (Figure 3A).

TAA stimulation also led to notable increases in the protein expressions of COX-2 and iNOS in the mouse liver (Figure 3B). However, following pre-treatment with G-Re, the protein levels of these inflammatory-related proteins, COX-2 and iNOS, in the mouse liver were significantly reduced in a dose-dependent manner compared to the model group.

TAA intervention also triggered the activation of the NLRP3 inflammasome, resulting in significant elevations in the protein expression levels of NLRP3 and caspase-1 p20 (Figure 3C), as well as the production of downstream cytokines including IL-18, and IL-1β. However, upon G-Re intervention, the expression levels of these inflammasome-related proteins exhibited a reversal of this effect.

#### 3.1.4 G-Re supressed autophagy activity in a TAA-induced liver injury mouse model

**FIGURE 4:**
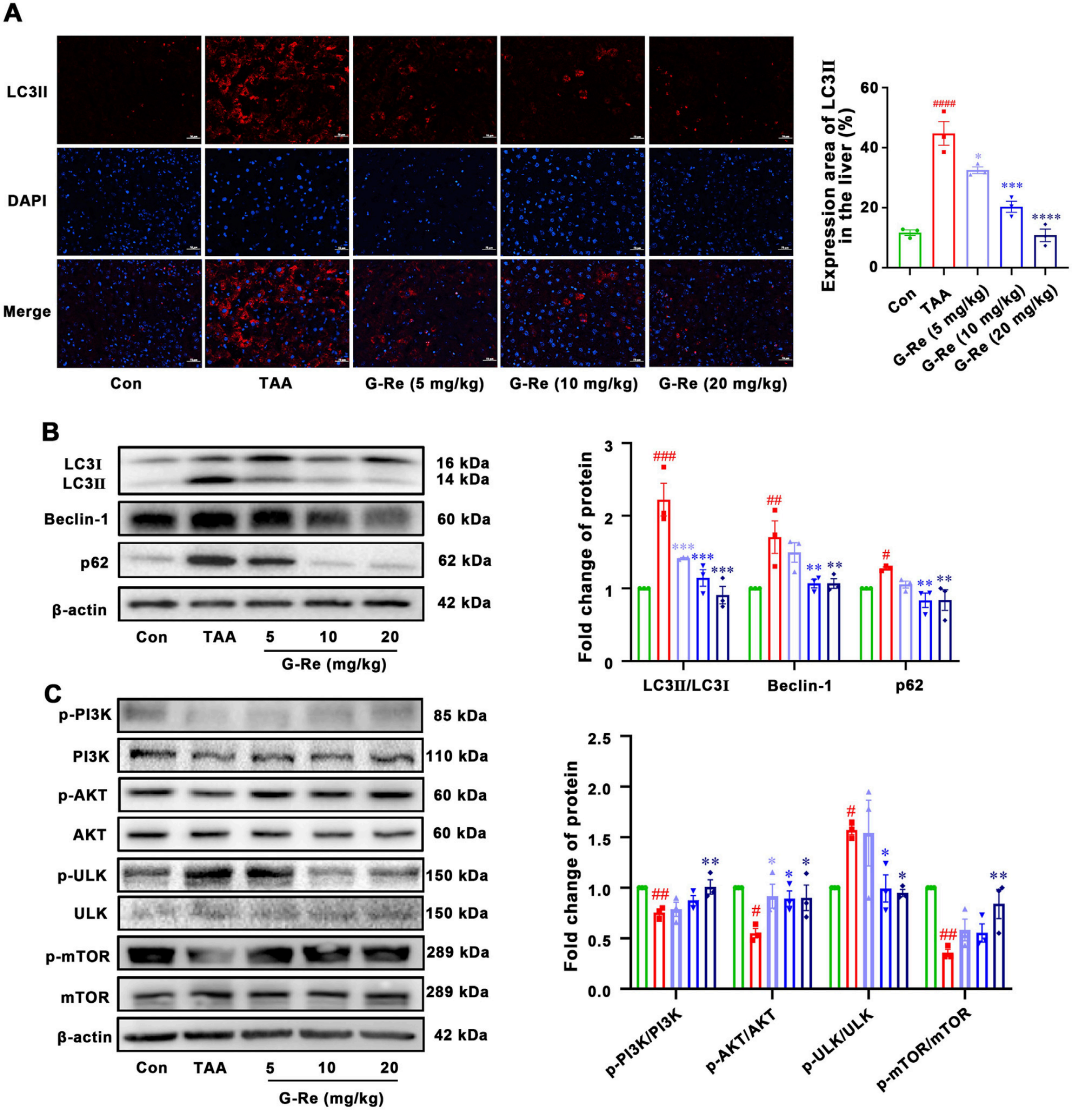
G-Re suppresses autophagy activity in mice with TAA-induced Liver Injury (n = 3). **(A)** Immunofluorescent staining of LC3II in liver tissues. Blue DAPI stain the nucleus and red fluorescence marks LC3II protein in the cytoplasm or nucleus. Scale bar = 50 μm. **(B)** Changes on autophagic proteins, including LC3I, LC3II, Beclin-1 and p62 expressions. **(C)** Changes on protein targets in the mTOR/PI3K/AKT cascade, including p-PI3K/PI3K, p-AKT/AKT, p-ULK/ULK, and p-mTOR/mTOR expressions. #*p* < 0.05, ##*p* < 0.01, ###*p* < 0.001 vs. Con group; *p < 0.05, **p < 0.01, ***p < 0.001 vs. TAA group.

In comparison to the control group, the TAA group exhibited a significant increase in both the area and intensity of LC3II fluorescence (Figure 4A). TAA also led to significantly higher LC3II/I ratios and increased levels of Beclin-1 and P62 compared to those in the control group (Figure 4B). This data suggested that intraperitoneal injection of TAA markedly elevated the autophagic level in mice. However, pre-treatment of G-Re in a dose-dependent manner reduced the fluorescence area and intensity of LC3II, as well as the protein expressions of LC3II/I, Beclin-1 and P62. This demonstrates that G-Re treatment effectively inhibited excessive autophagy.

To elucidate the underlying mechanism of how G-Re potentially modulated autophagy activity, the molecular targets within the PI3K/Akt signaling pathway were investigated. TAA stimulation decreased the phosphorylation levels of PI3K and Akt (Figure 4C). However, pre-treatment with G-Re increased the phosphorylation levels of PI3K/AKT signaling cascade. Furthermore, in comparison to the model group, G-Re elevated the phosphorylation level of mTOR and reduced the phosphorylation level of ULK. This suggested that G-Re may suppress autophagy levels by activating the PI3K/Akt signaling cascade.

### 3.2 *In vitro* study

#### 3.2.1 Anti-inflammatory effects of G-Re on LPS-induced HSC-T6 cells

**FIGURE 5:**
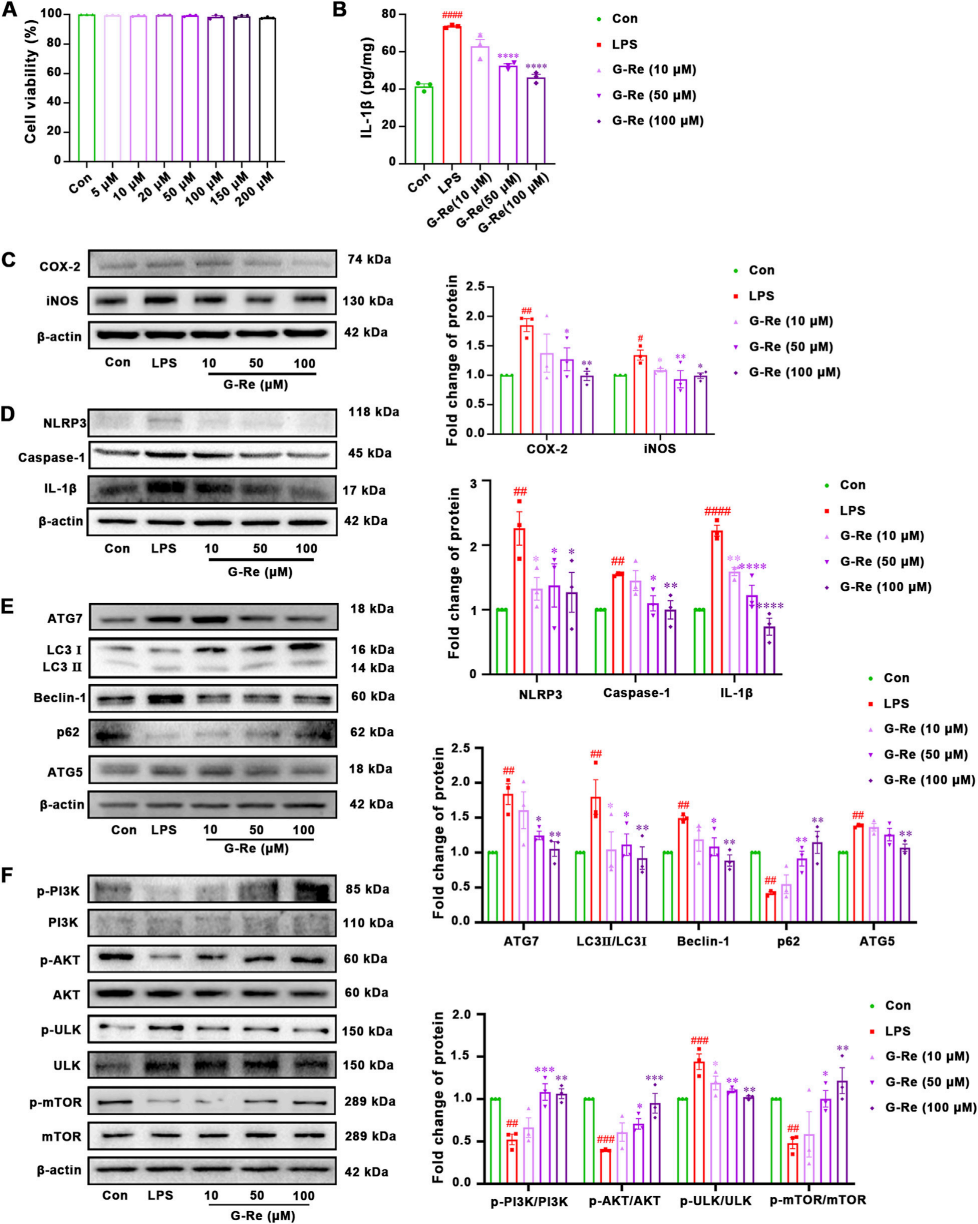
G-Re exhibited anti-inflammatory effect and suppressed autophagy activity on LPS-induced HSC-T6 cells (n = 3). **(A)** Cell viability of HSC-T6 cells. **(B)** The changes on IL-1β levels. **(C)** Changes on COX-2 and iNOS protein expressions. **(D)** Changes on NLRP3, Caspase-1 and IL-1β protein expressions. **(E)** Changes on autophagic proteins, including ATG7, LC3I, LC3II, Beclin-1, p62, and ATG5. **(F)** Changes on protein targets in the mTOR/PI3K/AKT cascade, including p-PI3K/PI3K, p-AKT/AKT, p-ULK/ULK, and p-mTOR/mTOR expressions. #*p* < 0.05, ##*p* < 0.01, ###*p* < 0.001 vs. Con group; *p < 0.05, **p < 0.01, ***p < 0.001 vs. TAA group.

The mechanisms underlying G-Re’s hepatoprotective effects were further explored in LPS-induced HSC-T6 cells. Firstly, G-Re showed no significant impact on HSC-T6 cell viability (*p* > 0.05) within a 200 μM concentration range (Figure 5A).

The anti-inflammatory effects of G-Re *in vitro* were consistent with those observed in the animal experiments. Compared with the control group, LPS stimulation significantly increased the level of IL-1β, and G-Re pretreatment dose-dependently reduced the IL-1β level (Figure 5B). In comparison to the control group, LPS stimulation significantly increased the protein expression levels of COX-2 and iNOS (Figure 5C). However, after pre-treatment with G-Re, the expression levels of COX-2 and iNOS decreased significantly.

LPS stimulation also triggered the activation of inflammasomes, resulting in a significant increase in the protein expression levels of NLRP3, caspase-1 p20, and IL-1β (Figure 5D). However, upon G-Re intervention, the expression levels of these inflammasome-related proteins exhibited a significant reversal.

#### 3.2.2 G-Re supressed autophagy activity on LPS-induced HSC-T6 cells

In comparison to the CON group, LPS significantly increased the LC3II/I ratio and the expression levels of Beclin-1, Atg5, and Atg7 (Figure 5E), while the expression level of P62 significantly decreased. These changes suggested that LPS elevated the autophagic activity in HSC-T6 cells. However, pre-treatment with G-Re reversed these alterations, suggesting that G-Re treatment effectively inhibited excessive autophagy levels.

Consistent with the *in vivo* findings, LPS stimulation for 12 h led to decreased phosphorylation levels of PI3K and AKT compared to the control group (Figure 5F). However, G-Re increased the phosphorylation levels of PI3K and AKT, suggesting that G-Re effectively activated the PI3K/Akt signaling cascade in LPS-stimulated HSC cells. Additionally, in comparison to the model group, G-Re increased the phosphorylation levels of mTOR and decreased the phosphorylation levels of ULK. This suggested that G-Re supressed autophagy, potentially by activating the PI3K/Akt signaling cascade.

#### 3.2.3 The correlation between autophagy and anti-inflammatory activities of G-Re in LPS-induced HSC-T6 cells

Our results suggested that G-Re inhibited autophagy levels and inflammatory responses in both TAA-induced acute liver injury mice and LPS-induced HSC cells. To further investigate the potential relationship between the anti-inflammatory effects of G-Re and its inhibition of autophagy levels, experiments with the mTOR inhibitor rapamycin (RAPA), both in the presence and absence of G-Re were conducted.

**FIGURE 6:**
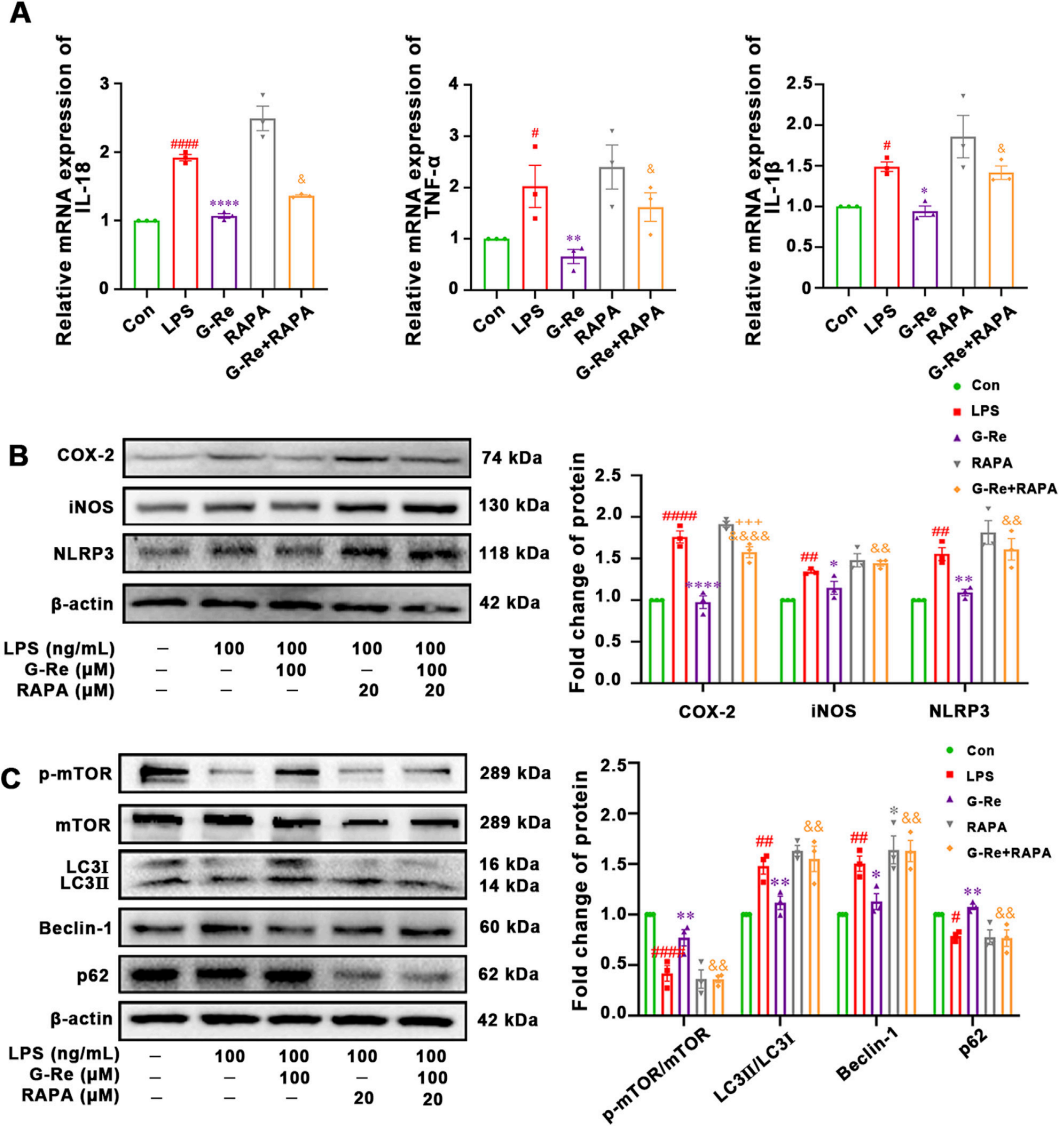
The correlation between autophagy and anti-inflammatory activities of G-Re on cells. (n = 3) **(A)** Changes on mRNA expressions of IL-18, TNF-α and IL-1β. **(B)** Changes on COX-2 and iNOS protein expressions. **(C)** Changes on protein targets in the mTOR/PI3K/AKT cascade and autophagic proteins, including LC3I, LC3II, Beclin-1, p62, and p-mTOR/mTOR expressions. #*p* < 0.05, ##*p* < 0.01, ####*p* < 0.0001 vs. Con group; *p < 0.05, **p < 0.01, ****p < 0.0001 vs. TAA group; &&*p* < 0.01, &&&&*p* < 0.0001 vs. G-Re.

Compared to the G-Re group, additional stimulation with RAPA in conjunction with G-Re for 12 h significantly increased the mRNA levels of IL-1β, IL-18, and TNF-α (Figure 6A). This result suggested that G-Re’s capacity to reduce the inflammatory factors such as IL-6, IL-18, and TNF-α was partially attenuated after restoring autophagy levels. Therefore, it is plausible that G-Re’s inhibitory effect on the inflammatory response is, at least in part, mediated by the suppression of autophagy expression.

Furthermore, the co-treatment of RAPA for 12 h significantly reversed the suppressed levels of NLRP3, COX-2, and iNOS (Figure 6B). This suggested that G-Re’s ability to inhibit the NLRP3 pathway and lower the expression levels of proteins like COX-2 and iNOS were also compromised when autophagy levels were reactivated.

In comparison to the G-Re group, additional stimulation with RAPA in combination with G-Re for 12 h significantly increased the expression levels of LC3II/LC3I and Beclin-1 (Figure 6C). Concurrently, it decreased the phosphorylation levels of mTOR and the expression level of P62. These results suggested that RAPA restored autophagy activity and attenuated G-Re’s inhibitory effect on autophagy levels.

#### 3.2.4 PI3K/AKT signaling-related mechanism of G-Re in suppressing autophagy

Our results showed that G-Re suppressed autophagy and the NLRP3 inflammasome, likely mediated by the PI3K/AKT cascade in TAA-induced acute liver injury mice and LPS-induced HSC cells. To confirm this, HSC-T6 cells were co-incubated with the PI3K autophagy inhibitor LY294002 and G-Re to assess the impact of PI3K/AKT inhibition on G-Re’s regulation of autophagy levels.

**FIGURE 7:**
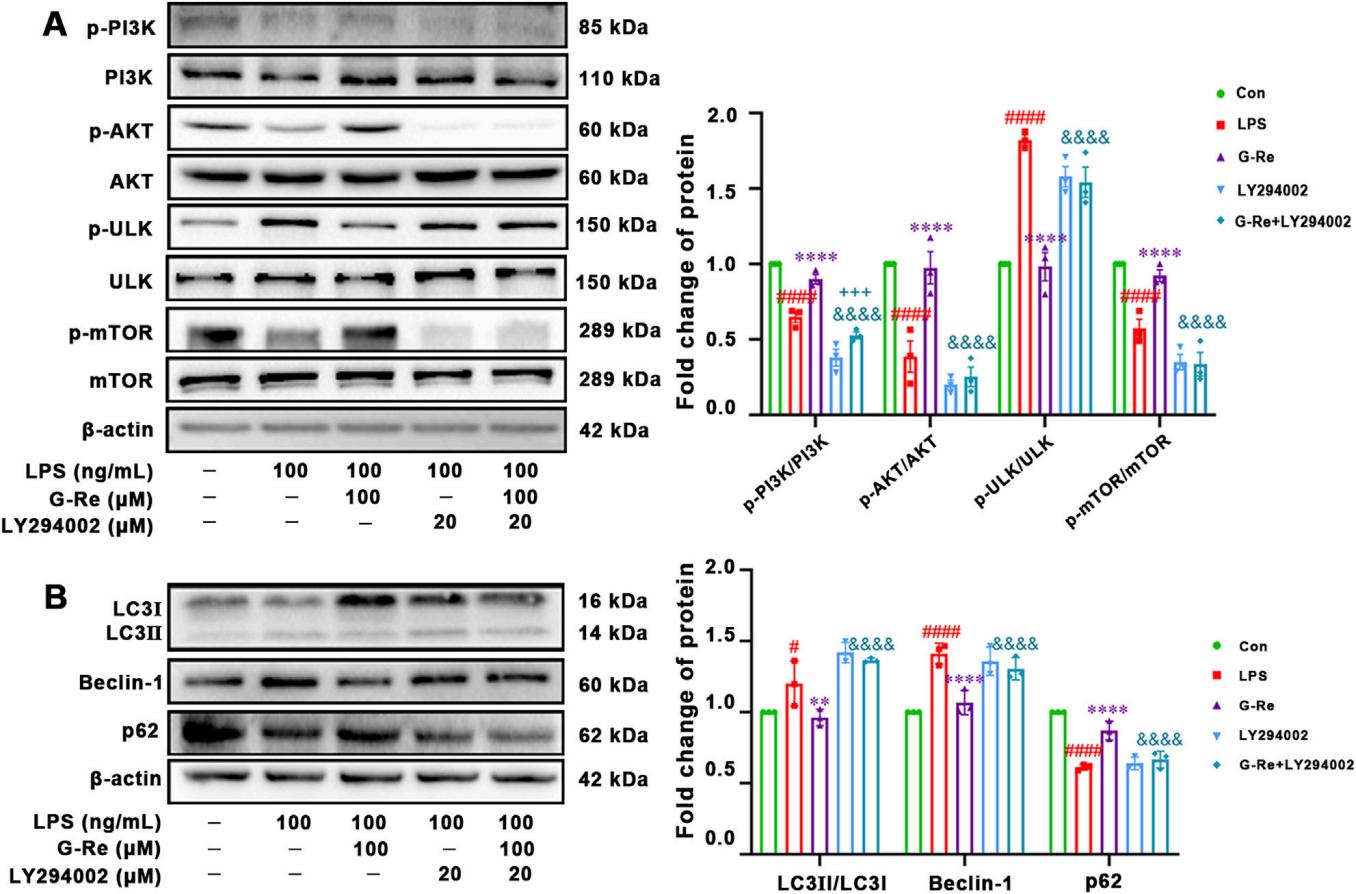
PI3K/AKT signaling-related mechanism of G-Re in suppressing autophagy **(A)** Changes on protein targets in the mTOR/PI3K/AKT cascade, including p-PI3K/PI3K, p-AKT/AKT, p-ULK/ULK, and p-mTOR/mTOR expressions. **(B)** Changes on autophagic proteins, including LC3I, LC3II, Beclin-1 and p62. #*p* < 0.05, ####*p* < 0.0001 vs. Con group; **p < 0.01, ****p < 0.0001 vs. TAA group; &&&*p* < 0.001, &&&&*p* < 0.0001 vs. G-Re.

The co-incubation of LY294002 and G-Re resulted in reduced phosphorylation levels of PI3K and AKT compared to the G-Re group (Figure 7A). This suggested that the PI3K inhibitor constrained G-Re’s ability to activate the PI3K/Akt signaling cascade. Furthermore, there was a significant increase in the phosphorylation levels of ULK and a decrease in the phosphorylation levels of mTOR in the combined G-Re and LY294002 group compared to the G-Re-only group. These findings suggest that inhibiting G-Re’s ability to activate the PI3K/Akt signaling cascade also hinders its capacity to suppress autophagy levels.

The co-incubation with LY294002 for 12 h significantly reduced the protein expression level of P62 while increasing the protein levels of LC3 and Beclin-1, compared to the G-Re group (Figure 7B). This further confirms that the suppression of G-Re’s ability to activate the PI3K/Akt signaling cascade led to the restoration of autophagy levels. Altogether, G-Re inhibited autophagy, at least partly, by activating the PI3K/Akt signaling cascade.

## 4 Discussion

TAA, alcohol, acetaminophen, and D-galactosamine-induced acute intoxication models have been proved well-established tools in studying the agents’ response to both acute and chronic injuries. TAA, as a hepatotoxicant agent, effectively induces liver damage by affecting key aspects of liver function, including protein synthesis, RNA, DNA, and Gamma-glutamyl transpeptidase activity (Sepehrinezhad et al., 2021; Akhtar and Sheikh, 2013). Using these widely accepted models for liver injury, our results suggested that G-Re treatment protected liver tissue by inhibiting inflammatory responses and reducing hepatic autophagy. Our data also suggested that the PI3K/AKT/mTOR-mediated mechanisms were the prominent pathways involved in the protective effects of G-Re on TAA-induced liver injury.

G-Re (C_48_H_82_O_18_), a ginsenoside derived from the roots of traditional Chinese medicinal plants, *Panax ginseng* C. A. Mey. or *Panax notoginseng* (Burk.) F.H. Chen, has exhibited a wide range of pharmacological activities. These included anti-diabetic properties, the alleviation of nervous system and cardiovascular diseases, anti-cancer effects, anti-viral activity, enhancement of sperm motility, and treatment of erectile dysfunction (Gao et al., 2022). However, research on the potential of G-Re in alleviating liver injury is limited. Our study revealed significant hepatoprotective properties of G-Re in multiple agent-induced acute liver injury models, as evidenced by a substantial reduction in central vein and sinusoidal congestion, as well as decreased inflammatory cell infiltration. Furthermore, G-Re demonstrated a dose-dependent reduction in the levels of AST and ALT, suggesting a prominent hepatoprotective effect. Notably, the effective dosages of G-Re (5–20 mg/kg) were lower than those of the positive control, DG (30 mg/kg), which has been extensively studied for its hepatoprotective effects on the liver (Shoala et al., 2021). Additionally, no obvious adverse reactions were observed in the animal experiments. In summary, G-Re showed the potential to be developed as a safe and effective therapeutic agent for treating liver injury. However, further investigation is needed to elucidate the exact role of G-Re in liver injury and the underlying mechanisms.

Previous studies have shown that G-Re possesses anti-inflammatory effects by reducing the levels of TNF-α, IL-6, prostaglandinE2, and nitric oxide induced by LPS. These effects have been linked to the suppression of AMPK and NF-κB signaling pathways (Quan et al., 2012). Consistent with these findings, our study demonstrated that G-Re exhibited dose-dependent anti-inflammatory activities against TAA-induced liver injury. G-Re effectively reduced the mRNA expressions of pro-inflammatory cytokines such as IL-6, TNF-α and IL-1β. Furthermore, G-Re decreased the protein expressions of COX-2 and iNOS, highlighting its broad anti-inflammatory activities.

G-Re’s ability to reduce mRNA expression of IL-1β has led to the extensive investigation into its modulation of the NLRP3 inflammasome. In TAA-induced liver injury, molecules released due to liver damage activate the NLRP3 inflammasome in immune cells. This inflammasome plays a crucial role in processing and releasing IL-1β, a potent pro-inflammatory cytokine that exacerbates liver inflammation and worsens the injury (Szabo and Csak, 2012). NLRP3 inflammasomes are well-known for initiating the caspase 1-dependent maturation of IL-1β and IL-18 precursor cytokines. They have been extensively studied and can detect a wide range of danger signals (Xu et al., 2018). Our findings suggested that G-Re reduced the protein expression of NLRP3, as well as caspase 1, IL-1β and IL-18 at the protein levels. This data suggested that G-Re’s anti-inflammatory activities in combating liver injury was associated with the suppression of NLRP3 inflammasome activation, which may help prevent the worsening of liver injury.

Previous studies have suggested that G-Re can regulate cellular autophagy activity, enhancing the survival of human CD4^+^ T cells (Son et al., 2010) and preventing 3-methyladenine-induced catagen phase acceleration (Jeong et al., 2023). Autophagy is a process of lysosomal degradation responsible for breaking down excess cytoplasm and dysfunctional organelles, such as mitochondria, to maintain cellular balance. Disruptions in autophagy can contribute to the development of various human diseases (Ni et al., 2012). Autophagy plays an important role in various aspects of liver health and liver-related conditions, including clearing misfolded proteins, regulating nutrient and energy levels, monitoring organelle turnover, and managing lipid balance. Deficiencies or inhibition of autophagy can lead to hepatocyte cell death, steatohepatitis, and hepatocellular carcinoma (Ni et al., 2012). Interestingly, previous studies have demonstrated that liver injury induced by substances like CCl_4_, or TAA can lead to elevated levels of autophagy in mice. Autophagic activity has also been detected in activated stellate cells within damaged human liver tissue. Impairment of autophagic function in cultured mouse stellate cells and in mice after injury resulted in reduced fibrogenesis and matrix buildup. This suggested that targeted reduction of autophagic activity within fibrogenic cells in the liver and other organs could potentially serve as a therapeutic approach for patients with fibrotic disorders (Hernández–Gea et al., 2012). In our present study, LPS-induced stellate cells (HSC-T6 cells) was established to further investigate the mode of action of G-Re on autophagy. Consistent with the previous study, LPS stimulation led to an increase in the LC3II/LC3I ratio, along with elevated p62 levels, suggesting an increased autophagic flux (Yang et al., 2019). Interestingly, G-Re exhibited moderate effects in normalizing LC3II/LC3I ratio and p62 levels. This suggested that G-Re’s ability to modulate autophagy may contribute to the alleviation of liver damage.

Dysregulation of autophagy has been implicated in many liver diseases, and it is now recognized that proper regulation of autophagy is key to the treatment of liver injury (Wang et al., 2019). Therefore, an in-depth study to understand the pathways through which G-Re regulates autophagy. The PI3K/AKT/mTOR pathways play interconnected and pivotal roles in autophagy regulation (Granato et al., 2017; Long and Pi, 2020). mTOR acts as a negative regulator of autophagy; and its activation suppresses autophagy. In conjunction with mTOR, the PI3K-AKT pathway modulates autophagy. Activation of AKT inhibits TSC1/2, leading to increased mTOR activity, which, in turn, suppresses autophagy. Conversely, AKT inhibition relives mTOR-mediated autophagy suppression, enabling autophagy to proceed. Our findings revealed that G-Re was dose-dependently restored the supressed activities of p-PI3K, p-AKT and mTOR, suggesting a suppression of autophagy. Thus, G-Re’s regulation of autophagy may involve the PI3K/AKT/mTOR pathway, which could contribute to its protective effects in liver injury.

Our study delved into the mechanisms through which G-Re protects the liver by supressing the NLRP3 inflammasome and autophagy in LPS-stimulated rat hepatic stellate cells (HSC-T6 cells). As expected, G-Re reduced inflammatory markers and the expression of NLRP3 inflammasome-related proteins. Interestingly, when we co-treated the cells with the mTOR inhibitor RAPA and G-Re, autophagy activity was restored, but inflammatory marker expressions were also reversed. This has led to the investigation of the interplay between autophagy and the NLRP3 inflammasome. Our results suggested that restored autophagy activity resulted in increased expressions of NLRP3 inflammasome and its downstream mediators, as well as higher expressions of TNF-α in liver stellate cells. While previous studies have shown that autophagy can inhibit the NLRP3 inflammasome, recent research has found that autophagy can also promote the NLRP3 inflammasome in some cases (Cao et al., 2019). For instance, under starvation conditions, autophagy can enhance caspase-1 activation through a non-classical pathway dependent on Atg5, leading to increased synthesis of IL-1β and IL-18 (Dupont et al., 2011). Our results showed that G-Re reduced LPS-induced ATG5 protein expressions, suggesting that the mechanism might be relevant to the regulation of autophagy and its modulation of the NLRP3 inflammasome. This study mainly used acute liver injury models, which may not fully reflect chronic liver disease progression in humans. While we identified the PI3K/AKT/mTOR pathway and NLRP3 inflammasome as key targets of G-Re, it may still not reflect the full molecular mechanisms as *in vitro* cell models may not capture the full complexity of liver tissue interactions. Finally, long-term safety and clinical efficacy of G-Re require future study. Further studies are needed to investigate the detailed mechanisms by which G-Re regulates Atg5-dependent autophagy and its impact on the NLRP3 inflammasome. Additionally, it would be interesting to explore whether changes in the NLRP3 inflammasome, in turn, affect autophagy activity.

## 5 Conclusion

In conclusion, our study has provided compelling evidence for the therapeutic potential of G-Re in protecting the liver from TAA-induced liver injury in C57BL/6 mice. G-Re effectively mitigated liver inflammation and supressed autophagic activity by modulating the PI3K/AKT/mTOR signaling pathway. Our *in vitro* experiments with liver stellate cells further revealed that the suppression of the NLRP3 inflammasome by G-Re was primarily mediated through the downregulation of autophagy activity. Collectively, our findings support the potential use of G-Re as a promising therapeutic agent for acute liver injuries, shedding light on the underlying mechanisms of its action.

## Data Availability

The original contributions presented in the study are included in the article/supplementary material, further inquiries can be directed to the corresponding authors.
